# Cerebellopontine angle cavernous hemangioma

**DOI:** 10.1016/S1808-8694(15)31056-9

**Published:** 2015-10-19

**Authors:** Mônica Porto Alves Alcantara, Paulo Roberto Lazarini, José C.E. Veiga, Erick S. Barboza, Carmen L.P. Lancellotti

**Affiliations:** 1 MD, otorhinolaryngologist under training at the Otorhinolaryngology Department of the São Paulo Santa Casa Hospital.; 2 PhD, Instructor Professor at the Medical School of the São Paulo Santa Casa Hospital, Assistant Physician at the Otorhinolaryngology Department of the São Paulo Santa Casa Hospital.; 3 PhD, Director of the Neurosurgery Department of the São Paulo Santa Casa Hospital.; 4 MD, neurosurgeon, former resident at the Neurosurgery Department of the São Paulo Santa Casa Hospital.; 5 PhD, Associate Professor at the Pathology Department of the São Paulo Santa Casa Hospital.

**Keywords:** cerebellopontine angle, differential diagnosis, cavernous hemangioma

## INTRODUCTION

Cavernous hemangioma, also known as cavernous angioma, accounts for 10-20% of vascular malformations.1,2 Extra-axial lesions are rare. Most tumors are found in the sinuses, Meckel’s cavity, posterior fossa, including the cerebellarpontine angle and internal auditory meatus.3

This paper reports the clinical case of a patient with cavernous hemangioma involving the internal auditory meatus whose initial diagnosis was vestibular schwannoma.

## CASE REPORT

JMM, female, 51 years of age, Caucasian, born in Pernambuco, complained of humming in her right ear and of slow, progressing hearing loss, with both conditions afflicting her for the past three years. She also experienced sporadic dizzy spells when turning to the left in episodes that lasted for less than a minute, usually followed by nausea and often accompanied by louder humming and hearing loss.

Audiometric tests pinpointed deep right-ear sensorineural hearing loss and mild left-ear hearing loss. Neuro-otologic tests suggested right-side vestibular deficit syndrome.

**Figure 1:**
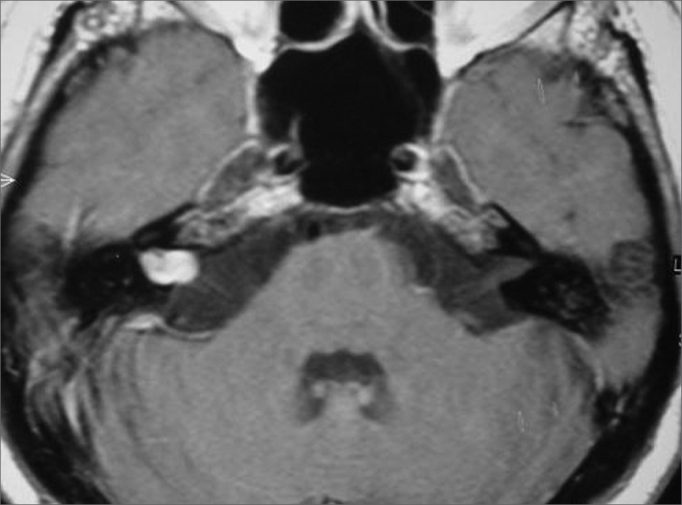
Skull MRI scan (axial T1 contrast-enhanced) showing right-side cavernous hemangioma.

No changes were found in the cranial CT scan, both with and without enhancement. Skull MRI images showed a 1.2x0.8 cm lobular structure expanding the right-side internal auditory meatus with a minor cisternal component, characterized by intermediate-level signal on T1, hypersignaling on T2 and impregnation by paramagnetic agent. (Figure 1)

The patient was submitted to a translabyrinthine approach on April 14, 2004. The procedure had to be aborted due to intense intra-op bleeding when opening the dura. The patient was re-operated on April 16, 2004. This time the suboccipital approach was chosen and the tumor was removed.

The pathologist’s report identified cavernous angioma with recent thrombosis.

## DISCUSSION

In this case report a patient with cavernous hemangioma in the internal auditory meatus came to our service complaining of auditory loss accompanied by humming and sporadic dizziness. The patient’s facial nerve was not involved. Even though some authors4,5 claim this type of tumor often involves the nerve, the literature review done by Babu et al.1 concluded that peripheral facial palsy is not a frequent finding.

Asymmetric auditory loss and vestibular examination suggesting vestibular deficit syndrome led us to consider the presence of a tumor in the retro-cochlear area later visualized in MRI. It is worth mentioning that MRI is more sensitive and more specific than CT1,3,5. In our case, CT scans found no abnormalities in spite of the hemangioma in the patient’s internal auditory meatus.

Differential diagnosis is important and includes mainly meningioma, vestibular schwannoma, facial nerve schwannoma2,3,5,6 and lipoma.1

According to Pappas et al.1,2, schwannomas present intermediate signaling on T1 and hypersignaling on T2 with gadolinium uptake, as described by the radiologist in our case.

## CONCLUSION

Cavernous hemangiomas in the internal auditory meatus are rare and may be characterized by unilateral sensorineural auditory deficiency. MRI is the technique of choice to achieve differential diagnosis. Final diagnosis can only be reached after pathology exam.
